# Manchette-acrosome disorders and testicular efficiency decline observed in hypercholesterolemic rabbits are recovered with olive oil enriched diet

**DOI:** 10.1371/journal.pone.0202748

**Published:** 2018-08-23

**Authors:** Layla Simón, Abi K. Funes, María A. Monclús, Regina Colombo, María E. Cabrillana, Tania E. Saez Lancellotti, Miguel W. Fornés

**Affiliations:** 1 IHEM, Universidad Nacional de Cuyo, CONICET, Mendoza, Argentina; 2 Instituto de investigaciones, Facultad de Ciencias Médicas, Universidad del Aconcagua, Mendoza, Argentina; Zhejiang University College of Life Sciences, CHINA

## Abstract

High-fat diet is associated with hypercholesterolemia and seminal alterations in White New Zealand rabbits. We have previously reported disorders in the development of the manchette-acrosome complex during spermiogenesis and decreased testicular efficiency in hypercholesterolemic rabbits. On the other hand, olive oil incorporated into the diet improves cholesterolemia and semen parameters affected in hypercholesterolemic rabbits. In this paper, we report the recovery—with the addition of olive oil to diet—from the sub-cellular mechanisms involved in the shaping of the sperm cell and testicular efficiency altered in hypercholesterolemic rabbits. Using morphological (structural, ultra-structural and immuno-fluorescence techniques) and cell biology techniques, a reorganization of the manchette and related structures was observed when olive oil was added to the high-fat diet. Specifically, actin filaments, microtubules and lipid rafts—abnormally distributed in hypercholesterolemic rabbits—were recovered with dietary olive oil supplementation. The causes of the decline in sperm count were studied in the previous report and here in more detail. These were attributed to the decrease in the efficiency index and also to the increase in the apoptotic percentage in testis from animals under the high-fat diet. Surprisingly, the addition of olive oil to the diet avoided the sub-cellular, efficiency and apoptosis changes observed in hypercholesterolemic rabbits. This paper reports the positive effects of the olive oil addition to the diet in the recovery of testicular efficiency and normal sperm shaping, mechanisms altered by hypercholesterolemia.

## Introduction

High-fat diet promotes increased serum cholesterol levels in adult male rabbits (New Zealand)[1–3]. As a consequence, several alterations are induced in sperm and seminal parameters. Among others, we have observed an increase in abnormally shaped spermatozoa, a decrease in the sperm progressive motility and a reduction in the number of spermatozoa in semen[1]. Along with these changes, an increase in sperm membrane cholesterol level is also detected[4]. Some of these alterations could have an origin during spermatogenesis or spermiogenesis at the seminiferous tubular level.

On the other hand, the Mediterranean diet has been associated with cholesterolemia reduction in men with metabolic syndrome[5] and in hypercholesterolemic rabbits (HCARDA)[6]. The main component of the Mediterranean diet, olive oil (OO), is related to this cholesterol-lowering effect[7, 8]. The morphological anomalies of spermatozoa and the reduction in the sperm number identified under hypercholesterolemic conditions could be reversed with the OO administration [6].

The final structure of the sperm is acquired after complex mechanisms that involve several steps along several stages[9]. In the rabbit, VIII stages corresponding to a disposition of the spermatogenic cells undergoing transformation from Golgi to acrosome have been described[10, 11]. Golgi apparatus provides several vesicles while acrosome is developed[12]. In crustacean, it has been described a new structure rich in microtubules, the acroframosome, which is involved in cargoes transport during the acrosomal morphogenesis [13–15].Acrosome grows attached to the nucleus by the acroplaxome[16]. At the distal acrosomal grown zone–implantation fossae-, a circumferential groove is observed. Above this groove a ring–marginal ring- rich in F-actin is located; and below, a ring conformed by a circumferential electron dense material -perinuclear ring- as depicted at electron microscopy micrographs. The perinuclear ring is coupled to a cytoskeleton structure rich in microtubules (manchette). The manchette pulls the rings towards the root of the flagellum while the diameter of the rings is reduced[17]. Simultaneously, the nuclear material is condensed. These structures and processes are involved in sperm head elongation [18, 19].

In our previous work, we reported the localization of actin, tubulin and ganglioside GM1 in the elongation area [20]. This particular localization could indicate that microtubules interact with a network of actin filaments that may be associated with membrane microdomains in the elongation area. Nevertheless, the effect of dietary olive oil in the sperm head elongation process is unknown.

Testicles generate less number of ejaculated sperm cells during spermatogenesis under high-fat diet but the sperm number is recovered when olive oil is added to diet [6]. The sperm number reduction is associated with a testicular efficiency decline in hypercholesterolemic rabbits[20]. However, testicular efficiency under the administration of olive oil remains unknown until now.

Two themes were combined in this work in order to obtain new knowledge on the effects of dietary lipids on testicles: the effects of high-fat diet on spermatogenesis and the recovery by the olive oil supplementation. Based on the results, we hypothesize that high-fat diet may exerts changes in the cytoskeleton structures involved in the shaping of the sperm cell. High-fat diet could also promote a decrease of the testicular epithelial proliferation accompanied by apoptosis. On the other hand, these alterations could be reversed with the addition of olive oil to the diet.

## Materials and methods

### Ethics statement

Animal studies described here were reviewed and approved by the animal care and use committee of School of Medicine, National University of Cuyo (Institutional Committee for Use of Laboratory Animals, IACUC- http://fcm.uncuyo.edu.ar/paginas/index/cicual); protocol reference number: 06_150702.

### Reagents

Unless otherwise stated, all chemicals and solvents of the highest grade available were obtained from Sigma (St. Louis, MO, USA) and Merck (Darmstadt, Germany).

Euthanyle: Product authorized by SENASA (http://www.senasa.gov.ar, government animal health regulation) was applied as euthanasic drug. Formula: 40 g pentobarbital /100 ml and 5 g diphenylhydantoin /100 ml (Brouwer laboratory S.A., Argentina).

Phosphate buffer saline (PBS): was prepared following the manufacturer instructions, dissolving one tablet in 100 ml of double distilled water, preparing 1X PBS solution containing 137 mM sodium chloride, 2.7 mM potassium chloride, and 10 mM phosphate buffer, pH: 7.4 (MP Biomedicals, California, USA).

Cow fat: First bovine juice locally named “Primer jugo bovino”. This commercial preparation (composed by 55% saturated fat, manufactured by Juan López y CIA.) correspond to specific regulation in the Argentina Alimentary Code (http://www.anmat.gov.ar/alimentos/codigoa/CAPITULO_VII.pdf; article 543, resolution 2012, 19.10.84).

Olive Oil (OO): virgin extra olive oil (Argentinean Arauco variety) was provided by the “Panel de Cata”–expert committee—of National University of Cuyo (http://www.fca.uncu.edu.ar/categorias/index/panel-de-cata-mendoza-de-aceite-de-oliva).

### Rabbit model

**Fig 1:**
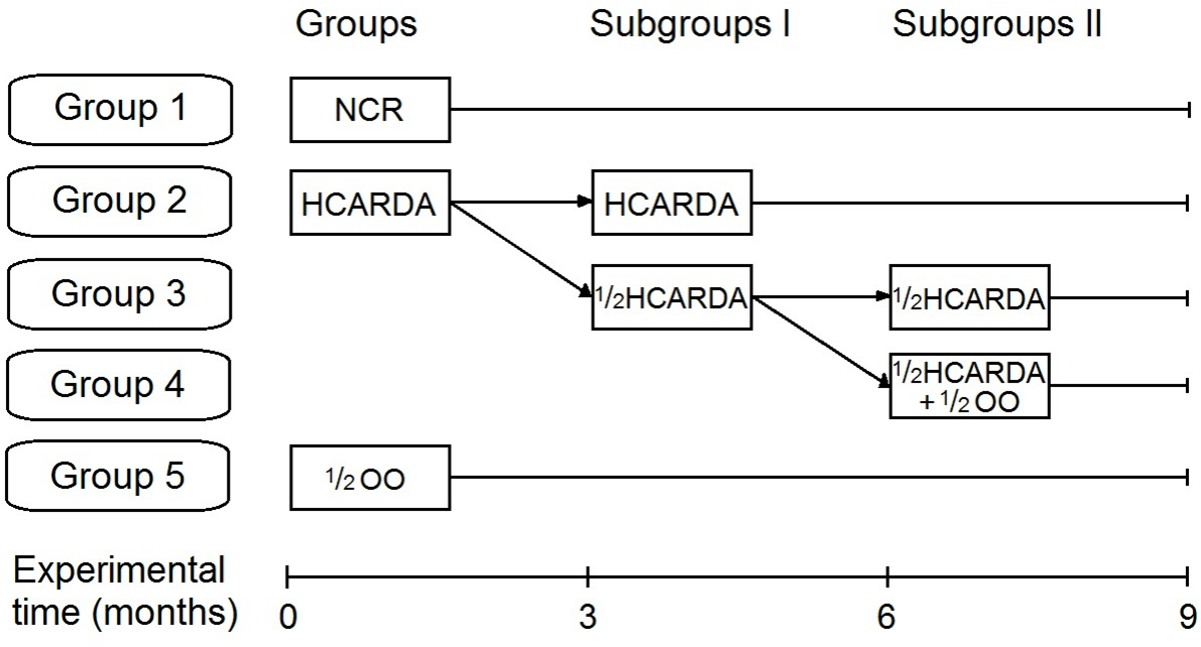
Feeding protocol and resulting groups. Adult rabbits were divided in three groups: normocholesterolemic rabbits were fed with balanced diet during 9 months (Group 1 = NCR), another group received high-fat diet (Group 2 = HCARDA), and the third group received olive oil diet (Group 5 = ½ OO). After 3 months, HCARDA was split in HCARDA (Group 2, Subgroup I) that continued with high-fat, and in ½ HCARDA that received half cow fat (Group 3, subgroup I = ½ HCARDA). Finally, ½ HCARDA was split again, one of them continued with half cow fat diet, and the other received half cow fat and half olive oil (Group 4, subgroup II = ½ HCARDA + ½ OO).

The rabbit model was set up following previous papers [1,6,20]. Briefly, adult male rabbits were fed ad libitum with a standard rabbit diet. This group of animals was named normocholesterolemic rabbits (NCR), in contrast to the hypercholesterolemic rabbits that were acutely fed with 14% v/w cow fat added to the standard diet. Other adult rabbits were fed with standard rabbit diet supplemented with virgin olive oil (½ OO, final concentration = 7% v/w, pellet saturation) to check any unexpected changes induced by OO administration. Fat was reduced and a fourth group was obtained feeding animals with half cow fat applied to the standard diet (½ HCARDA, Subgroup I). Finally, ½ HCARDA was also supplemented with OO (½ HCARDA + ½ OO, subgroup II) (Fig 1).

### Tissue collection

Lethal doses of pentobarbital (1 ml/5 kg; Euthanyle®) via pinna marginal veins were applied to rabbits. Then, whole testicles were surgically isolated. Testicles were placed on Petri dishes with PBS, and small cubes (2 x 2 mm) were cut and fixed for light and electron microscopy (see below). The remaining tissue was decapsulated with scissors and seminiferous tubules were transferred to a fresh PBS buffer containing collagenase (see below).

### Cholesterolemia

Blood samples were obtained fortnightly from marginal ear vein with heparinized syringes. Immediately after bleeding, blood was centrifuged at 1,100 x*g* by 10 minutes in a clinical centrifuge. Supernatant was carefully aspirated and aliquots were processed using GTlab kit (GTlab, Rosario, Argentina) to determine cholesterol levels.

### Semen assays, measurements and morphology

By applying an artificial vagina, ejaculated semen was collected monthly from all experimental conditions [21]. Semen samples were stored at 37°C, and analyzed for volume, aspect, pH, sperm motility, viability and concentration, as previously stated[1]. Total number of spermatozoa per ejaculate was calculated in semen samples from the last two months (experimental time). Remaining semen sample was washed twice in PBS, centrifuged 10 minutes at 750 x*g* and the final pellet was resuspended in fixative solution (4% paraformaldehyde in PBS). Then, smears of fixed sperms were stained with conventional Giemsa stain and morphological abnormalities were tabulated. Normal and abnormal forms of sperm were classified according to the type of sperm head alteration or the relation between head and tail sperm axis.

### Spermatogenic cells isolation

After euthanasia, testicles were obtained, tunica albuginea was removed with sterile forceps, and seminiferous tubules were treated with collagenase for 10 minutes at 37°C (5 mg/ml of collagenase, SIGMA C5138). Using a trans-illumination method, cycles of spermatogenesis were detected[22]. Sections of seminiferous tubules, developing the Golgi-acrosome complex in stages VI to VIII, were selected and cut under stereoscope [10]. The tubules were placed on slides containing 40 μl PBS and extruded with a cover-slip in order to obtain the spermatogenic cells. Spermatogenic cells were fixed and smeared on slides or transferred to microtubes (dx.doi.org/10.17504/protocols.io.rwid7ce).

### Asymmetry index measurement

**Fig 2:**
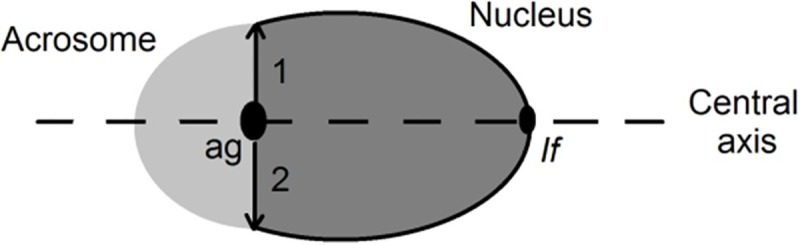
Asymmetry measurement. The light grey semicircle delineates the acrosome. The grey oval, partially covered by the acrosome, represents the nucleus. The central axis (dashed line) is an imaginary line that crosses the acrosomal granule (ag) and the implantation fossae (if): tail to head connection. Distances 1 and 2 are represented by arrows between the central axis and each acrosomal edge.

Spermatogenic cells were stained with Toluidine Blue (1 g dissolved in 100 ml of PBS) for 60 seconds and observed by light microscopy. After washing with PBS, the asymmetry in the development of the acrosome was studied in two measurement steps. First, the distances between the central axis and each acrosomal edge were established (distances 1 and 2, Fig 2). Secondly, the difference between distances 1 and 2 was calculated and related to the total distance between the two acrosomal borders. This relation was represented as a percentage. When distances 1 and 2 are similar, the asymmetry index approaches 0 (asymmetry index formula). Measurements were performed by Image J free software (http://imagej.nih.gov/ij/).

$$Asymmetry\;index=\frac{1-2}{1+2}\times 100$$

### Oil red O staining

Smears of fixed spermatogenic cells were washed with PBS and incubated 5 minutes with 60% isopropanol. Cells were covered with 0.5% Oil Red O (ORO) solution during 5 minutes (Oil Red O saturated solution in isopropanol:water, 3:2, generous gift from Barbieri`s lab, FIU, Miami, USA). ORO excess was eliminated with repeated water washings. Lipid droplets, stained with ORO were counted in three different samples from each condition and expressed as number of droplets per cell. Representative photomicrographs are shown for NCR, HCARDA and ½ HCARDA + ½ OO conditions.

### Optical microscopy

Histological analyses were performed following standard procedures. Small pieces of testicle were fixed with 4% formaldehyde, dehydrated in ethanol-xylene and embedded in paraffin. Five μm thickness sections were obtained on a sliding microtome, mounted on slides, de-waxed with xylene and stained with Toluidine Blue. Slides were examined by light microscopy (Nikon Eclipse 80i). Spermatogenic cells were identified following cell morphology patterns. Percentages of each cell types -from spermatogonia to elongated spermatids- were calculated (see testicular efficiency measurement).

### Transmission electron microscopy

Small pieces of testicle were fixed with 4% paraformaldehyde (w/v), 4% glutaraldehyde and 20% picric acid (v/v) saturated in PBS and post-fixed with 1% OsO4 (w/v) overnight at 4°C[23]. Then tissues were dehydrated using ethanol-acetone solvents. Dehydrated tissues were embedded in epoxy resin (Epon 812, Pelco, USA). Ultra-thin sections were obtained using ultra-cut equipment (Leitz), stained with uranyl acetate and lead citrate, and examined with a Zeiss EM 900 microscope (Zeiss, Oberkochen, Germany).

### Immuno-fluorescence staining

Fixed spermatogenic isolated cells were washed with PBS and permeabilized with 0.1% Triton X-100 (Sigma, T8532). Unspecific antigenicity was blocked with 2% BSA (Fraction V, Sigma, A8022). Then, cells were incubated in darkness with different markers alone or combined: antibody against α-tubulin (1:50, MP Biomedicals, 691251) detected with the secondary antibody conjugated with biotin (pan-specific antibody, Vector, PK7800) and avidin-fluorescein complex (Vector, SA5001); antibody against actin conjugated with Cy3 (3 μg/ml, Sigma, C6198, generous gift from Lopez`s lab, IHEM, Mendoza, Argentina); and cholera toxin subunit β conjugated with Alexa fluor 594 (5 μg/ml, MP Biomedicals, C22842) or fluorescein (Sigma, C1655). These markers were applied to identify–respectively- microtubules, actin filaments, GM1-riched lipid rafts and nuclear material. After washing, cells were mounted with Fluoroshield (Sigma, F6182) and examined in inverted microscope, NIKON TE2000.

### Western-Blot analysis

Proteins were extracted following established protocols by Sheng [24]. Testicles were dissected and homogenized with HEPES, KCl, EDTA, sucrose, spermine, spermidine, DTT and protease inhibitors (Sigma, P8340). After freeze centrifugation in Beckman Optima TLX, the supernatant was used to determinate the protein concentration. 40 μg of protein were separated by electrophoresis in acrilamide gels. Tubulin expression was evaluated using antibody against α tubulin (MP, 0869125). Histone H3 was used as a control (SC, 8656-R). Image J software was applied in order to determinate the intensity of bands obtained via Western-Blot analysis.

### Testicular efficiency measurement

Total spermatogenic cells per tubular seminiferous section were quantified. In addition, different types of spermatogenic cells were calculated and expressed as percentages of the total number of cells counted. Proliferation and differentiation efficiency rates were also obtained. Proliferation efficiency rate (*per*) was calculated dividing the number of ejaculated sperms by the percentage of spermatogonia in the seminiferous tubules:

$$per=\frac{ejaculated\;sperms}{spermatogonia}$$

Differentiation efficiency rate (*der*) was calculated dividing the number of ejaculated sperms by the percentage of spermatids:

$$der=\frac{ejaculated\;sperms}{spermatids}$$

*per* and *der* indexes for NCR were used as a standard parameter (control condition) and plotted as 100%.

### Apoptosis

Fixed spermatogenic isolated cells were washed with PBS and permeabilized with 0.1% Triton X-100 in PBS. Cells were assayed for apoptosis using TUNEL technique (TUNEL technology, In situ cell death detection kit, 11684795910, Roche). Cells were incubated 60 minutes at 37°C in darkness with TUNEL reaction mix. TUNEL reaction mix was prepared adding 1 part of enzyme solution to 9 parts of label solution. Propidium iodide was also applied to identify nuclear material (1 μg/ml, Sigma, P4864). After washing, cells were mounted with Fluoroshield (Sigma, F6182) and examined using inverted microscope NIKON TE2000. TUNEL-positive cells were quantified and expressed as a percentage of the total number of cells counted.

### Statistical analysis

Unless otherwise expressly noted, results were reported as means ± SD of at least three independent experiments. Differences between groups were evaluated by ANOVA test, followed by LSD Fisher test, considering a *p* value of less than 0.05 as statistically significant.

## Results

### Cholesterolemia

Serum cholesterol levels in rabbits reached values similar to those of our previous work (NCR: 25 ± 4 mg/dl; HCARDA: 87 ± 12 mg/dl; ½ HCARDA + ½ OO: 44 ± 10 mg/dl; ½ OO: 35 ± 10mg/dl; means ± SD) [6]. Rabbits on a high-fat diet developed hypercholesterolemia (*p*< 0.05) and when olive oil was added to the high-fat diet, cholesterol levels returned to normal.

### Seminal assays: Sperm concentration and morphological analysis

**Fig 3:**
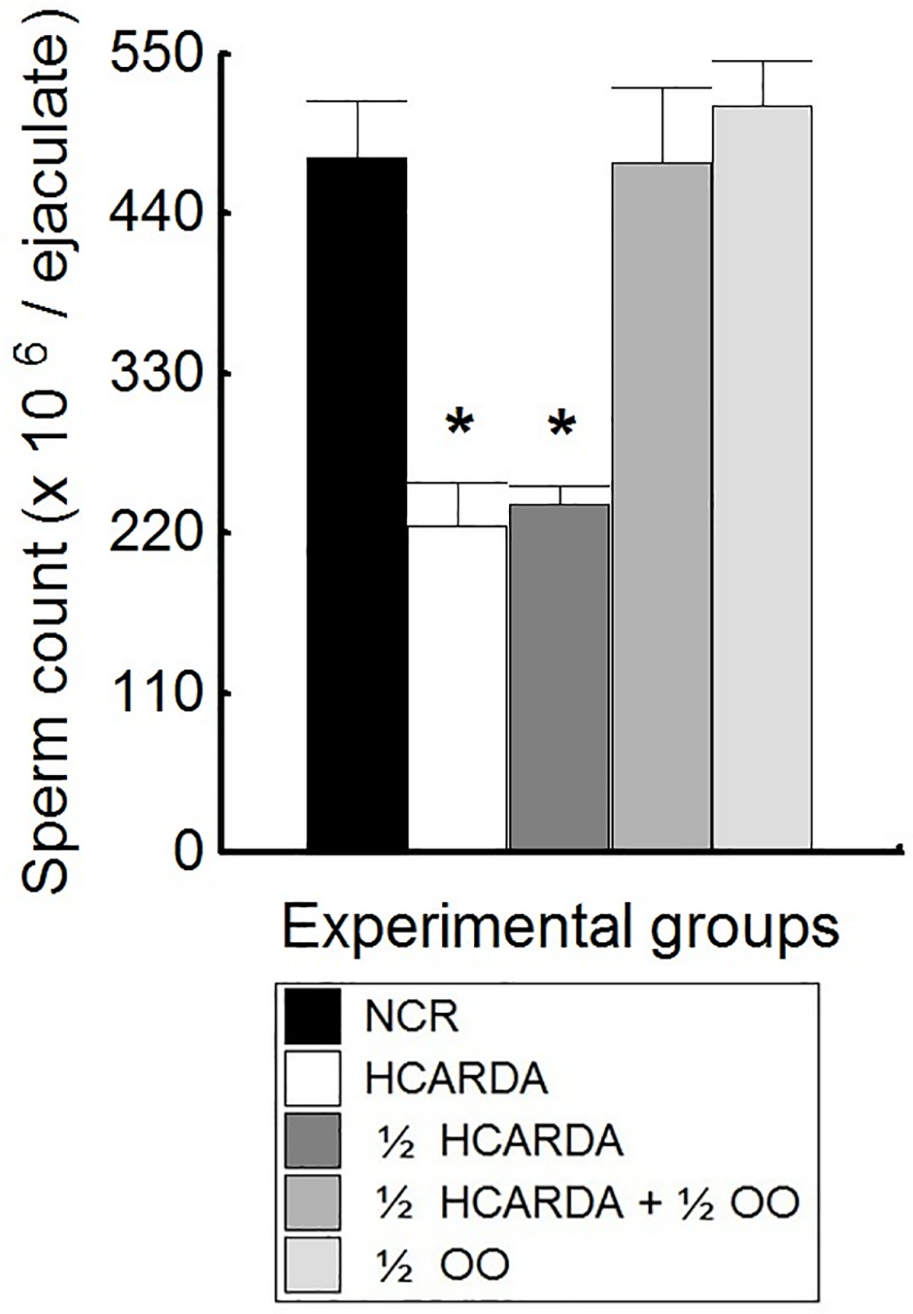
Total sperm count (million per ejaculate). Means of sperm cell number and SD from different feeding conditions were plotted: black bar represents NCR, white bar = HCARDA, dark grey bar = ½ HCARDA, grey bar = ½ HCARDA + ½ OO, and light grey bar = ½ OO. n = 10 samples per condition. Asterisks = *p*< 0.05.

Seminal parameters showed similar values to our previous report (data not shown) [6,20]. In this work, the total sperm count of the semen was specifically counted (Fig 3). These results showed a decrease in the number of spermatozoa under hypercholesterolemic conditions that was recovered after reduction in fat and supplementation with OO (Fig 3).

**Fig 4:**
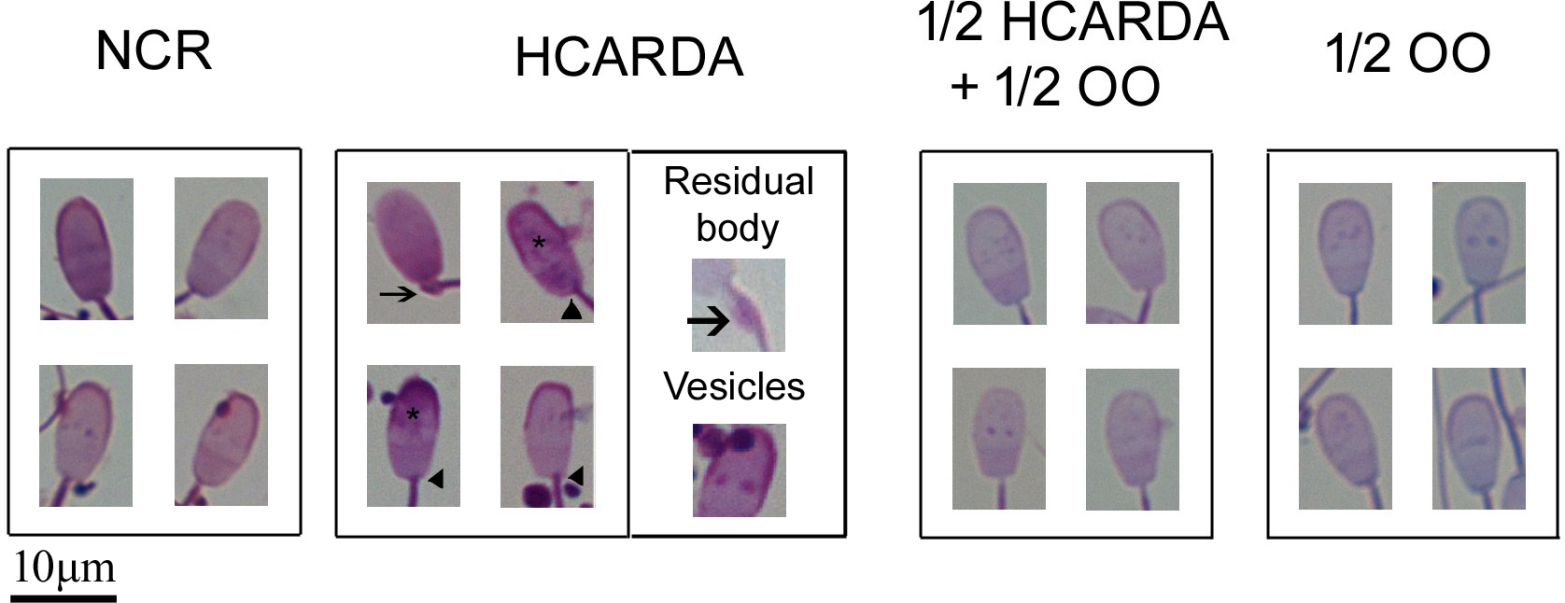
Sperm cell representative pictures from different experimental conditions. In the HCARDA group, the presence of residual body around the middle piece (arrow), several vesicles in the acrosome (asterisks) and an asymmetric tail implantation (arrowhead) were observed. Other experimental conditions did not present the same alterations and had normal morphology. The box in HCARDA corresponds to images of residual bodies and vesicles with larger magnifications.

The morphological analysis was carried out studying spermatozoa by optical microscopy. The most frequent sperm abnormalities are shown in Fig 4. In the spermatozoa of HCARDA it was observed: residual body (arrow), vesicles in the head of the spermatozoa (asterisks) and abnormal flagellum implantation (arrowheads, HCARDA, Fig 4). When diet was supplemented with olive oil, head and tail defects were reduced (½ HCARDA + ½ OO and ½ OO, Fig 4).

**Fig 5:**
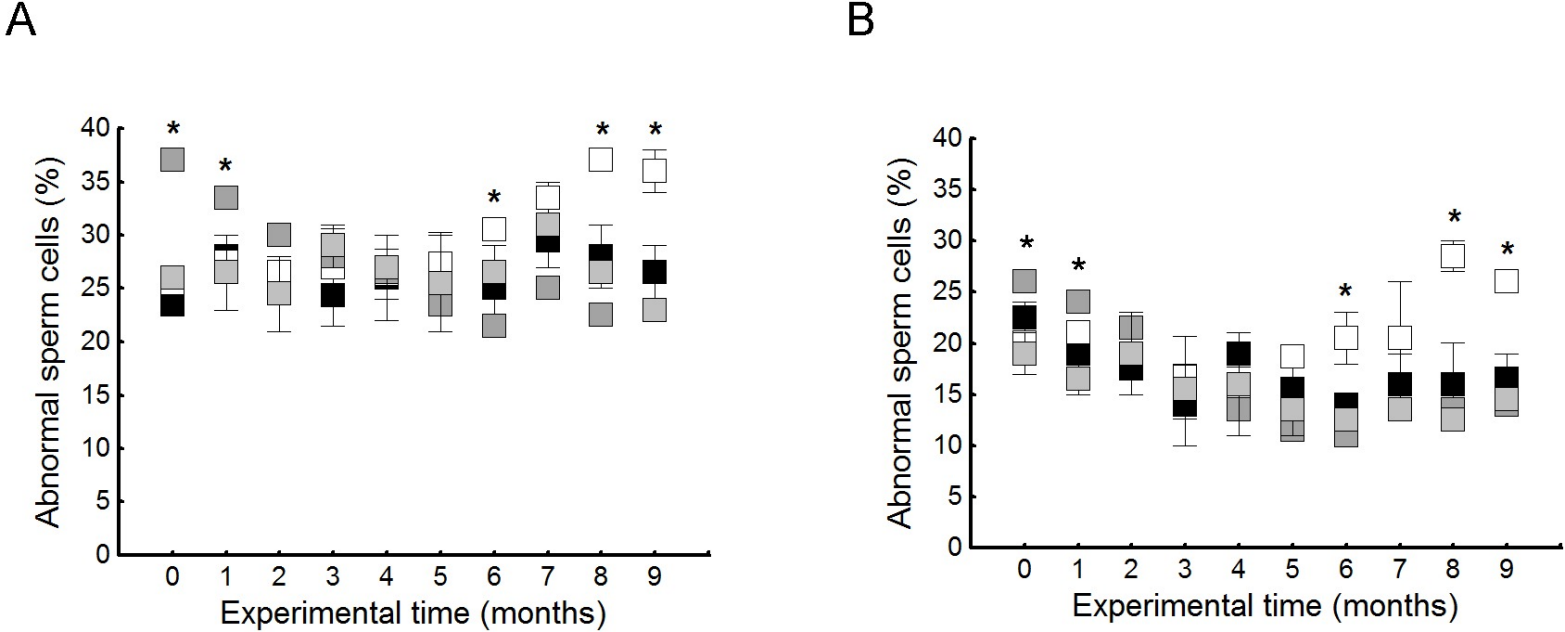
Percentage of sperm abnormalities under normal and experimental diets during 9 months. (A) Sperm cells with head defects (acrosomal lost, acrosomal vesicle presence, or tapered head) were quantified and tabulated as a percentage. (B)Sperm cells with abnormal tail implantation were quantified and tabulated as a percentage. Black squares: NCR, white squares: HCARDA, grey squares: ½ HCARDA + ½ OO and light grey squares: ½ OO. n = 30 cells per condition analyzed monthly. Asterisks = *p*< 0.05.

Sperm abnormalities were quantified in semen samples for 9 months (experimental time). Abnormalities were classified in A: sperm head abnormalities–described in Fig 4, HCARDA–, and B: sperm tail implantation abnormalities–sperm with the flagella implanted out of the central axis–. Hypercholesterolemia was consistent with an increase in abnormal sperm form after 6 months of experimental diet (HCARDA, Fig 5A). On the other hand, supplementation with olive oil allowed the recovery of normal sperm morphology after 3 months of experimental diet (½ HCARDA + ½ OO, Fig 5A). A frequent anomaly was the asymmetric tail implantation (implantation of the tail outside the sperm central axis; HCARDA, Fig 5B). When olive oil was added to the diet, alterations in the tail position disappeared (½ HCARDA + ½ OO, Fig 5B).

### Isolated spermatogenic cells

**Fig 6:**
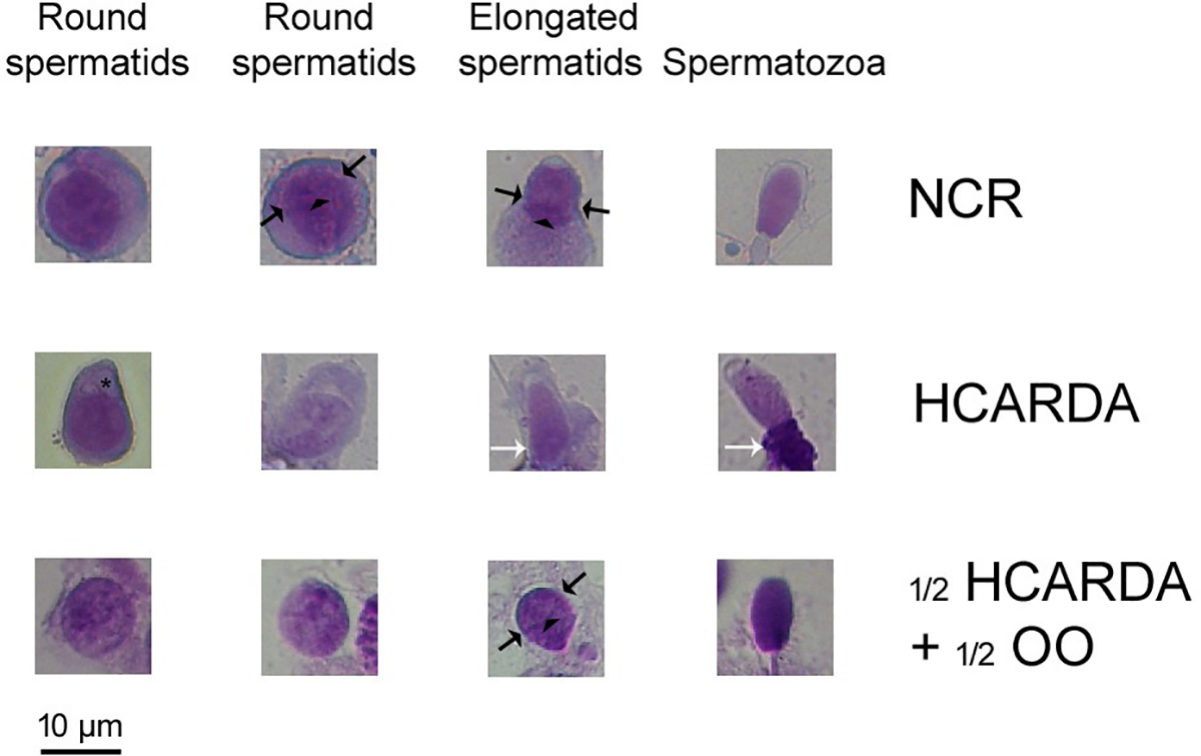
Morphology of cells isolated from seminiferous tubules. Spermatogenic cells are ordered according to the acrosomal stage from left (immature) to right (mature). Rows correspond to experimental groups: NCR (upper row), HCARDA (middle row) and ½ HCARDA + ½ OO (bottom row). Note the symmetric position of acrosomal edge (opposite black arrows) and the perinuclear ring (arrowhead) in cells from NCR and ½ HCARDA + ½ OO in contrast with the HCARDA. Also note abnormalities in flagella, like residual body (white arrows), and the presence of droplets (asterisk) in the acrosome zone in HCARDA group. 450X, Toluidine Blue stain.

To identify the mechanisms involved in the morphological alterations of spermatozoa, spermatogenic cells were analyzed. After extrusion of seminiferous tubules, spermatogenic cells were obtained. The cells were fixed, stained, photographed and classified by morphological criteria. Cells were ordered, from round spermatids—initial acrosome stages—to elongated spermatids and spermatozoa. This sequence made it possible to compare the same cell types under different experimental conditions. Some changes shown previously in hypercholesterolemic rabbits[20] were also observed in this work. HCARDA spermatogenic cells (Fig 6, middle row) were characterized by the asymmetric development of acrosomes, several droplets (asterisk) and thickening of the middle piece of the flagellum (white arrows). Instead, under olive oil incorporation, abnormalities decreased markedly (Fig 6: ½ HCARDA + ½ OO–bottom row).

### Measurement of the acrosomal asymmetry

**Fig 7:**
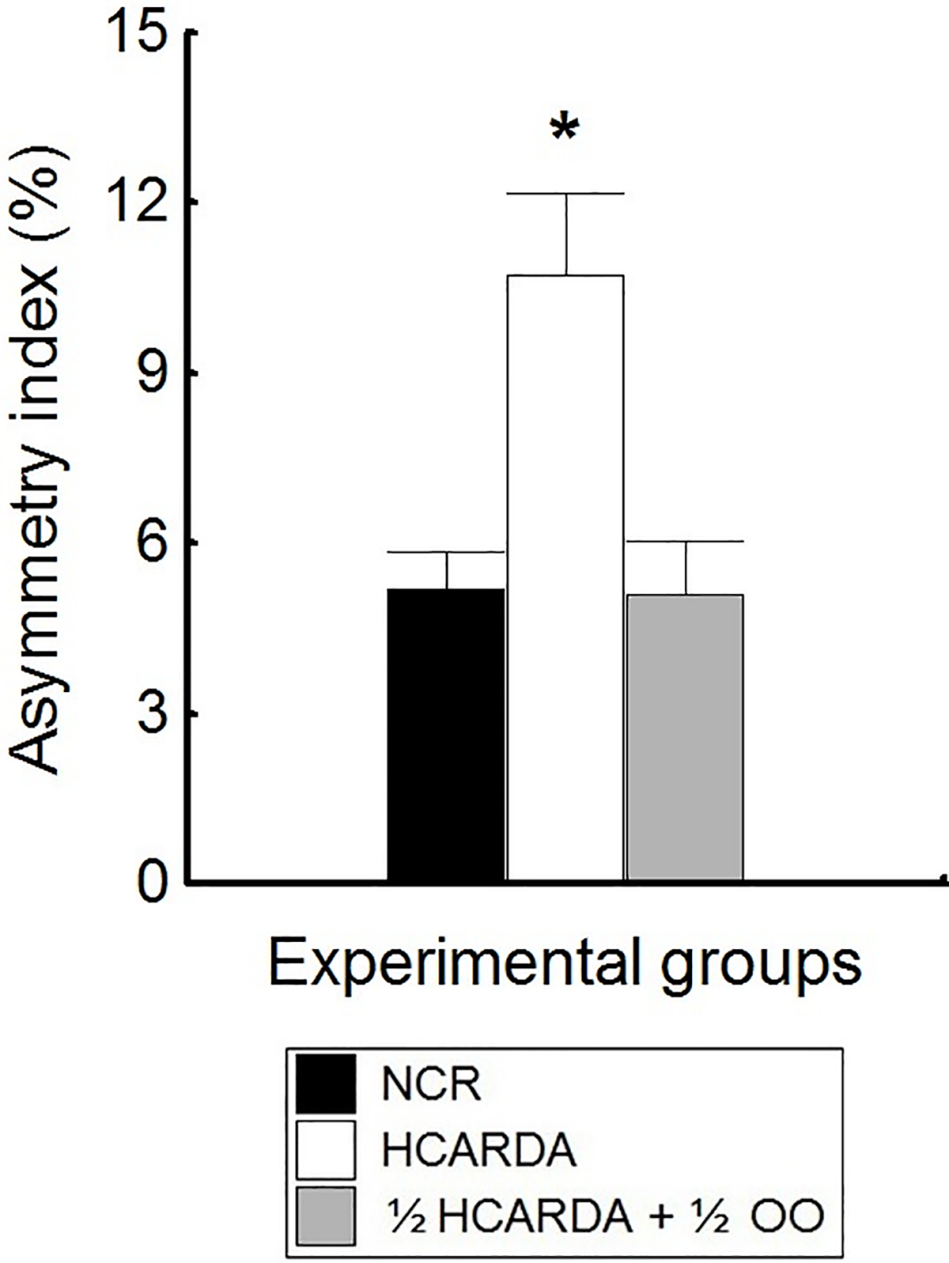
Acrosomal asymmetry measurement. Asymmetry index is expressed as a percentage and represented by bars (means ± SD). Black bars: NCR, white bars: HCARDA, and grey bars: ½ HCARDA + ½ OO. n = 30 cells per experimental group. Asterisk = *p*< 0.05.

The asymmetric development of the acrosome was depicted by the asymmetry index. When a high-fat diet was applied (HCARDA), the asymmetry index increased to approximately 10% (see materials and methods). On the other hand, when olive oil was added (½ HCARDA + ½ OO), the index changed to about 4% and overlaps with that of NCR (Fig 7).

### Structural and ultra-structural testicular changes

**Fig 8:**
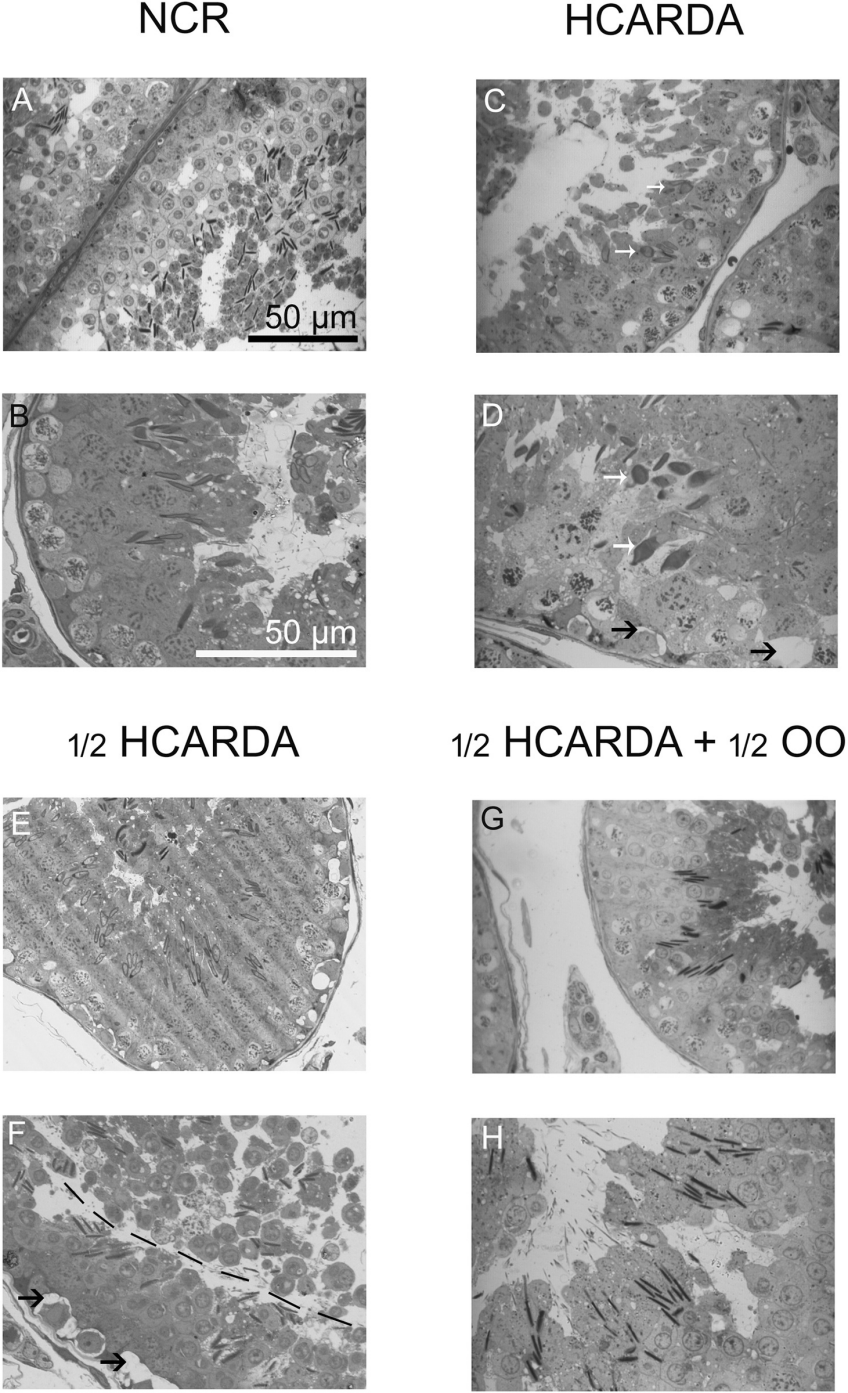
Light microscopy of semi-thin sections. Testis isolated from normal (NCR), hypercholesterolemic (HCARDA), half fat (½ HCARDA), and protected rabbits (½ HCARDA + ½ OO). White arrows point to abnormal sperm heads (HCARDA, pictures C and D). Black arrows point vesicles (HCARDA, picture D, and ½ HCARDA, picture F). Segmented line points epithelium detachment (½ HCARDA, picture F). Note the similarities between pictures A and B (NCR) with G and H (½ HCARDA + ½ OO). Magnifications: 400X (A, C, E and G, scale bar in A = 50 μm) and 620X (B, D, F and H, scale bar in B = 50 μm).

In the testicular sections, observed under a light microscope, we were able to recognize specific cellular dispositions of the seminiferous epithelium (Fig 8, pictures A and B, NCR). In HCARDA, different stages could also be observed because spermatogenesis was preserved, but the last steps of spermiogenesis were defective (abnormal sperm head were highlighted with white arrows in Fig 8, pictures C and D, HCARDA). However, when the proportion of fat was reduced (½ HCARDA, Fig 8, pictures E and F) the abnormal cells disappeared but some alterations remained (epithelium detachment observed in the F image). Only when olive oil was added to the diet, spermatogenesis was recovered (Fig 8, pictures G and H, ½ HCARDA + ½ OO).

**Fig 9:**
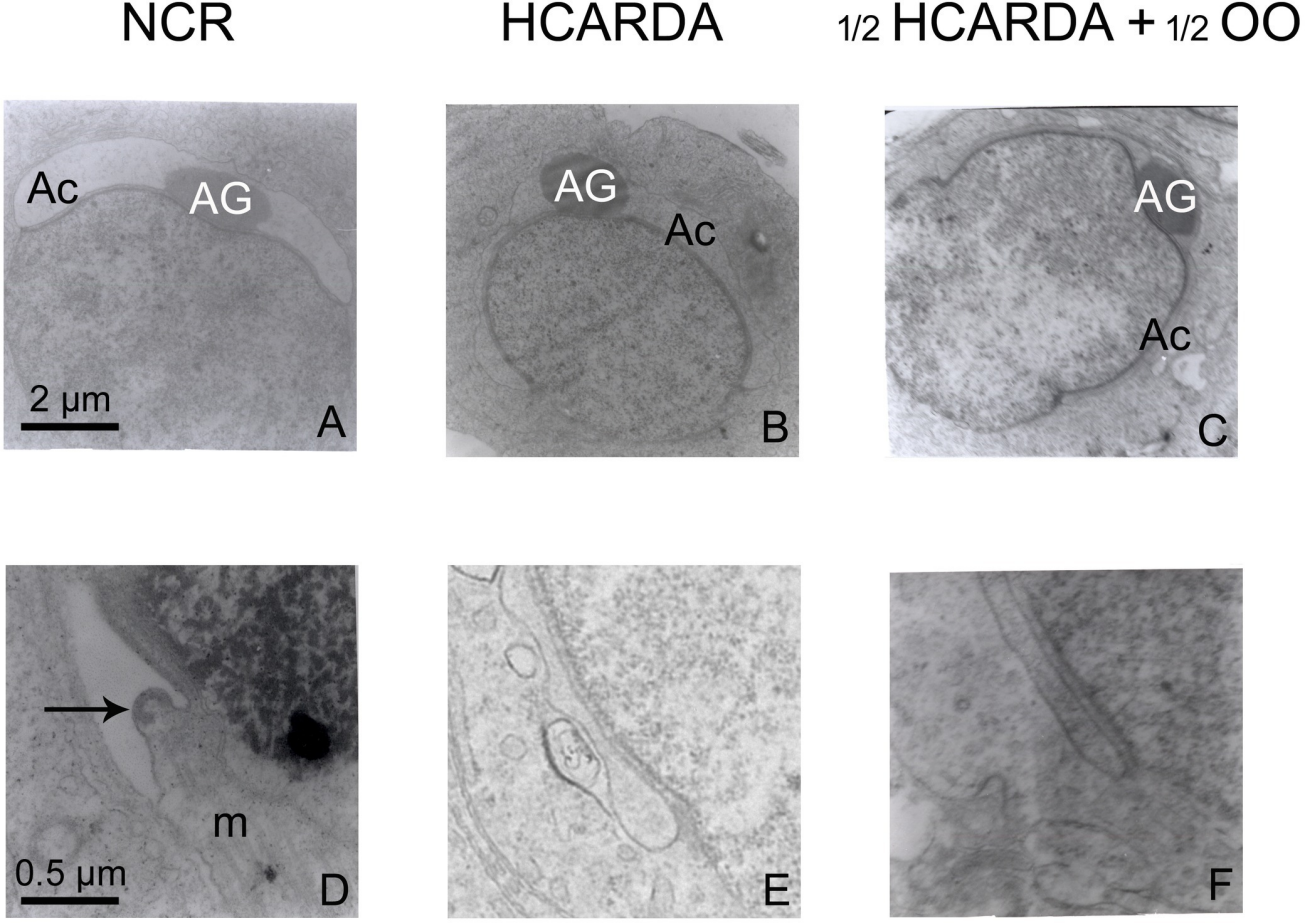
Ultra-structure of round spermatids. NCR (left column, A and D): Acrosome (Ac), acrosome granule (AG) located symmetrically from both acrosomal borders, perinuclear ring (arrow) and microtubules (m). HCARDA (middle column, B and E): No detection of acrosomal symmetry, perinuclear or manchette arriving to the ring. Moreover, lax and asymmetric acrosome (B), and several vesicles and whorl (E) could be observed under HCARDA condition. ½ HCARDA + ½ OO (right column, C and F): recovered acrosome ultra-structure and symmetry without vesicles or whorls. Magnification: 10.000X (A, B and C, scale bar in A = 2 μm) and 40.000X (D, E and F, scale bar in D = 0.5 μm).

Under ultra-structural definition, the transformation from Golgi to acrosome was clearly altered in the HCARDA group (Fig 9). In rabbits with normal nutrition, the acrosomal granule (AG) was observed equidistant from both acrosomal edges (Fig 9, NCR, A). In addition, several microtubules (m) were observed reaching the perinuclear ring (arrow, Fig 9, NCR, D). On the other hand, the high-fat diet promoted the abnormal development of acrosome in several cells, showing an asymmetrically positioned acrosomal granule (AG) relative to the acrosomal borders (Fig 9, HCARDA, B) with absence of the perinuclear ring and microtubules in the proximity of the acrosomal border. In addition, there were several vesicles and whorls close to the acrosome and the circumferential groove (Fig 9, HCARDA, E). When the diet was supplemented with olive oil, the acrosome (Ac) acquired a symmetric distribution around the acrosomal granule (AG) (Fig 9, ½ HCARDA + ½ OO, C). Furthermore, the inner and outer acrosomal membranes were close and parallel to the nuclear membrane, and no vesicles or whorls were detected (Fig 9, ½ HCARDA + ½ OO, F).

### Lipid droplets accumulation

**Fig 10:**
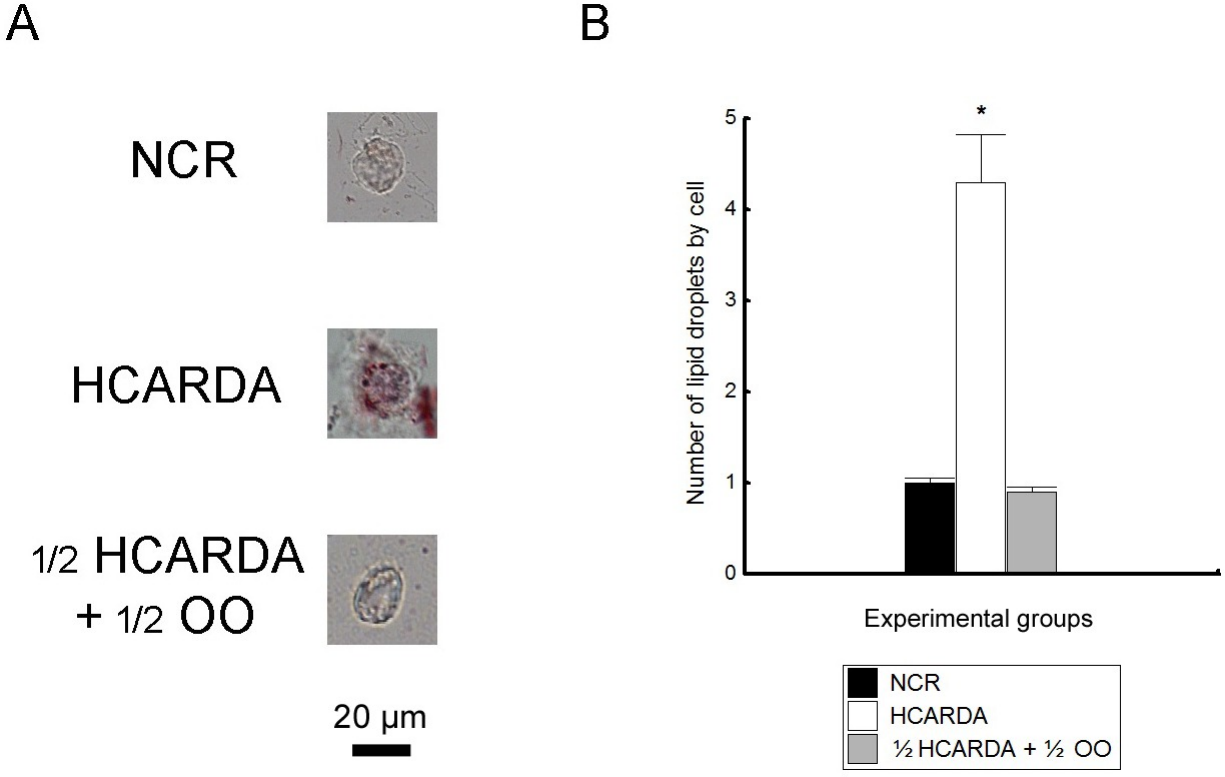
Lipid droplets detection. (A) Lipid droplets stained by ORO in NCR, HCARDA and ½ HCARDA + ½ OO. Only cells isolated from the testicles of the HCARDA experimental condition showed more than one drop. Magnification: 620X. (B) ORO positive lipid droplets for each cell were plotted and represented in black (NCR), white (HCARDA) and grey bars (½ HCARDA + ½ OO). n = 3 rabbits per experimental group. Asterisk = *p*< 0.05.

The lipid droplets—highlighted with oil red O—were clearly recognized in cells isolated from seminiferous tubules of HCARDA males, compared to the NCR and ½ HCARDA + ½ OO groups (Fig 10A). Number of ORO droplets by cell was increased under high-fat diet compared with the other experimental condition (Fig 10B).

### Manchette-acrosome development during spermiogenesis

Changes observed under light and transmission electron microscopy—at seminal and testicular levels—could be associated with modifications in the elongation complex of the sperm head. In this sense, we have reported alterations in microtubules, actin filaments and lipid rafts distribution in HCARDA rabbits [20], but there are no reports of the effects of dietary olive oil supplementation.

**Fig 11:**
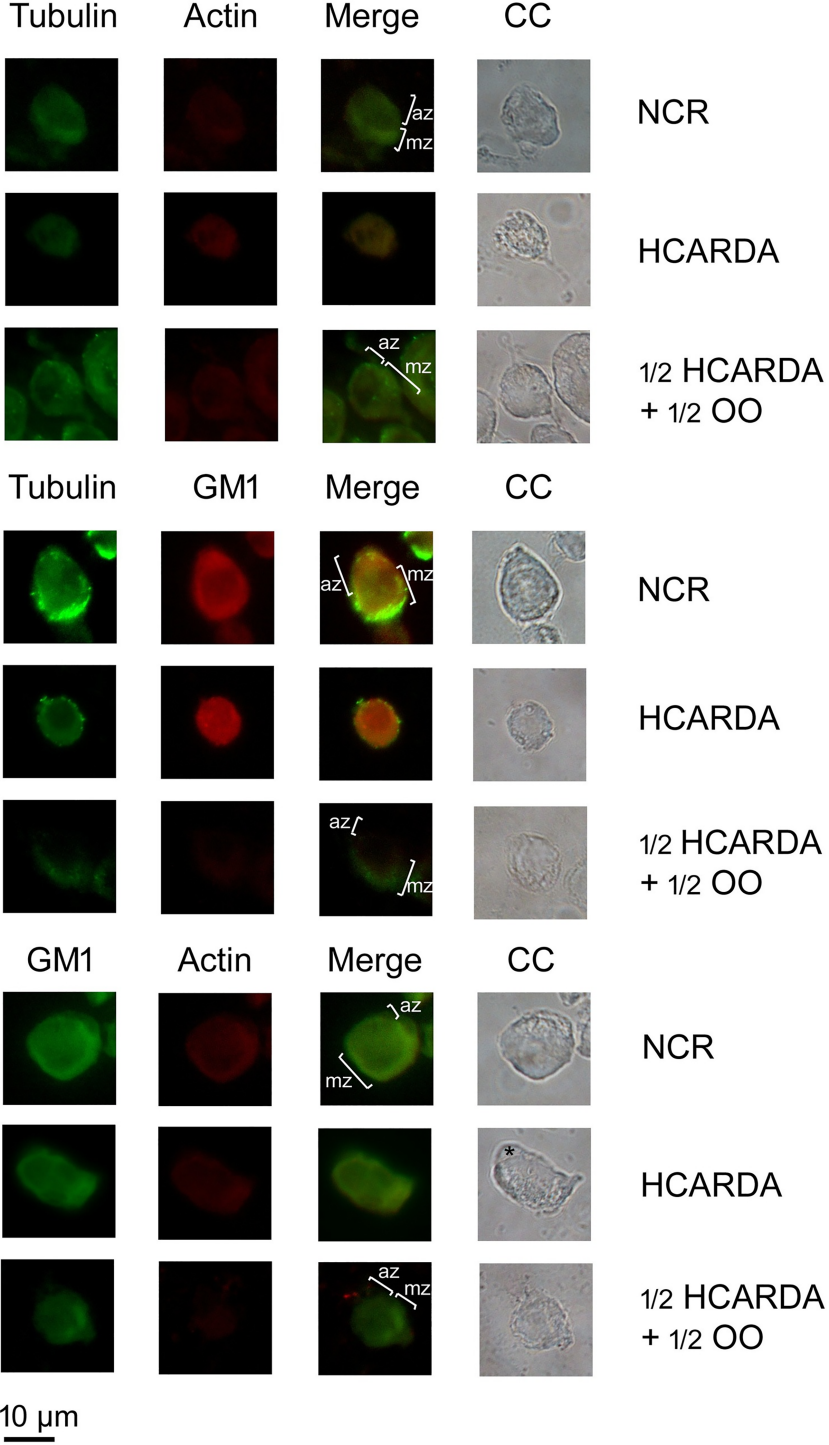
Microtubules, actin filaments and lipid rafts arrangement during spermiogenesis. Spermatogenic isolated cells were analyzed to test the components of the sperm head elongation complex. The microtubules of the manchette were detected using alpha-tubulin antibody and secondary antibody combined with FITC (tubulin columns). The actin filaments were stained with actin antibody conjugated with Cy3 (actin columns). GM1-enriched lipid rafts were detected using cholera toxin conjugated with Alexa flour 594 (GM1 column, red signal) or FITC (GM1 column, green signal). The combination of green and red colors (merge columns) and phase contrast images were also included (CC columns). In NCR, microtubules, actin filaments and GM1 co-localized and were distributed in the manchette zone (mz) opposed to the acrosome zone (az). In HCARDA, it was not possible to detect manchette or acrosome zone. Microtubules, actin filaments and GM1 were equally distributed. Also, an asymmetrical acrosome was observed (asterisk). In ½ HCARDA + ½ OO, cells were polarized and manchette and acrosome zone were detected. Magnification: 620X.

We studied the localization of manchette microtubules, actin filaments and GM1-enriched lipid rafts by immuno-fluorescence staining. In NCR, spermatogenic isolated cells were polarized. Microtubules, actin filaments and lipid rafts were distributed in the manchette zone (mz, Fig 11, NCR rows) opposite to the acrosome zone (az). Instead, when high-fat diet was administrated, cells lost their polarization (Fig 11, HCARDA rows). Microtubules, actin filaments and lipid rafts were detected homogeneously distributed in the cytoplasm of cells under hypercholesterolemic condition. In addition, these cells had an acrosome localized asymmetrically (asterisk). On the other hand, when olive oil was added to the high-fat diet, cells polarization was partially recovered (Fig 11, ½ HCARDA + ½ OO rows). The microtubules, actin filaments and lipid rafts were re-distributed, showing a co-localization in the manchette zone (mz).

Analyzing spermatids in previous stages of the spermiogenesis, the perinuclear ring was present in NCR and ½ HCARDA + ½ OO, but did not appear in HCARDA experimental condition (S1 Fig, white arrows). Moreover, actin filaments and microtubules were distributed in acrosomal and manchette zones in NCR and ½ HCARDA + ½ OO. On the other hand, cytoskeleton structures were poor in HCARDA spermatids (S1 Fig). Moreover, tubulin fluorescent intensity seemed to reduce under the high-fat diet but to increase with the olive oil supplementation (S1 Fig, tubulin column). Meanwhile, the actin fluorescent intensity showed similarities under different experimental conditions (S1 Fig, actin column). This result was confirmed studying tubulin protein expression via Western-blot (S2 Fig).

### Testicular efficiency

**Fig 12:**
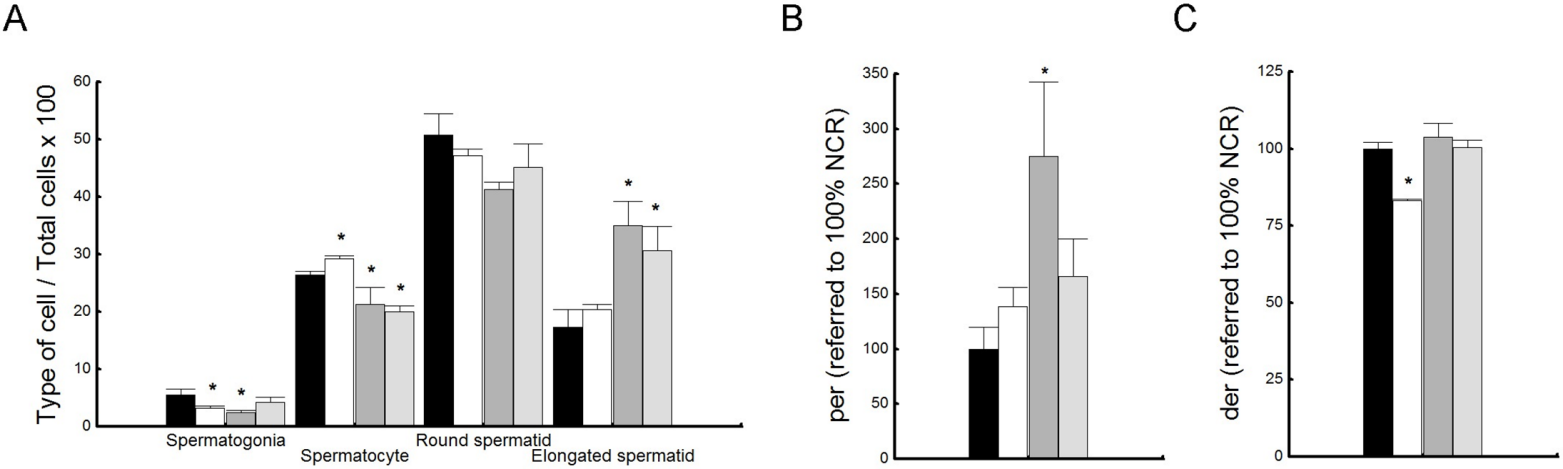
Testicular efficiency. (A) Percentages of spermatogenic cells by tubular seminiferous section. Each specific type of spermatogenic cell (spermatogonia, spermatocyte, round and elongated spermatid) present in a cross section of seminiferous tubules were counted and plotted as a percentage of the total cells counted. Mean ± SD of percentages of different spermatogenic cells were plotted for all experimental conditions: NCR = black bars, HCARDA = white bars, ½ HCARDA + ½ OO = grey bars, and ½ OO = light grey bars. (B) Proliferation efficiency rate (*per*) normalized to NCR. *per* was calculated dividing the number of sperm cells by the spermatogonia percentage. (C)Differentiation efficiency rate (*der*) normalized to NCR. *Der* was calculated dividing the number of sperm cells by the spermatid percentage. Mean ± SD of *per* and *der* were plotted using the same color code. n = 200 cells per condition in six separated experiments. Asterisks = *p*< 0.05.

The efficiency of spermatogenesis was calculated as the number of each type of spermatogenic cells per tubular seminiferous section. Spermatogenic cells were classified as spermatogonia, spermatocyte and round and elongated spermatids. These different types of cells were expressed as percentages of the total number of cells counted (Fig 12A).HCARDA rabbits showed a significantly low percentage of spermatogonia, but a higher percentage of spermatocytes compared to NCR (Fig 12A, HCARDA: white bars)[20]. There were no significant differences in spermatids percentages between HCARDA and NCR. On the other hand, when olive oil was added to the diet, the percentage of spermatogonia and spermatocytes decreased, meanwhile the percentage of elongated spermatids increased (Fig 12A).

The efficiency in the proliferation was estimated with the proliferation index (*per*). This index was calculated dividing the number of ejaculated sperm by the percentage of spermatogonia in the seminiferous tubules. As we previously reported, there was no a statistically significant difference between NCR and HCARDA in the proliferation efficiency rate (*per*, Fig 12B)[20]. When olive oil was added, *per* increased (Fig 12B) showing a recovery in proliferation efficiency with higher number of sperm cells without raising the spermatogonia percentage (Fig 12A).

Moreover, the efficiency in the differentiation was estimated with the differentiation index (*der*). This index was calculated dividing the number of ejaculated sperm by the percentage of spermatids in the seminiferous tubules. Differentiation efficiency rate was significantly lower in HCARDA compared to NCR and ½ HCARDA + ½ OO (*der*, Fig 12C). In this case, the high-fat diet affected differentiation efficiency, demonstrated by a reduction in sperm count, although no significant change in the percentage of spermatids was detected (Fig 12A). However, differentiation efficiency was restored by adding olive oil to the diet, with an increase in the percentage of spermatids and sperm cells (Fig 12A).

### Apoptosis

**Fig 13:**
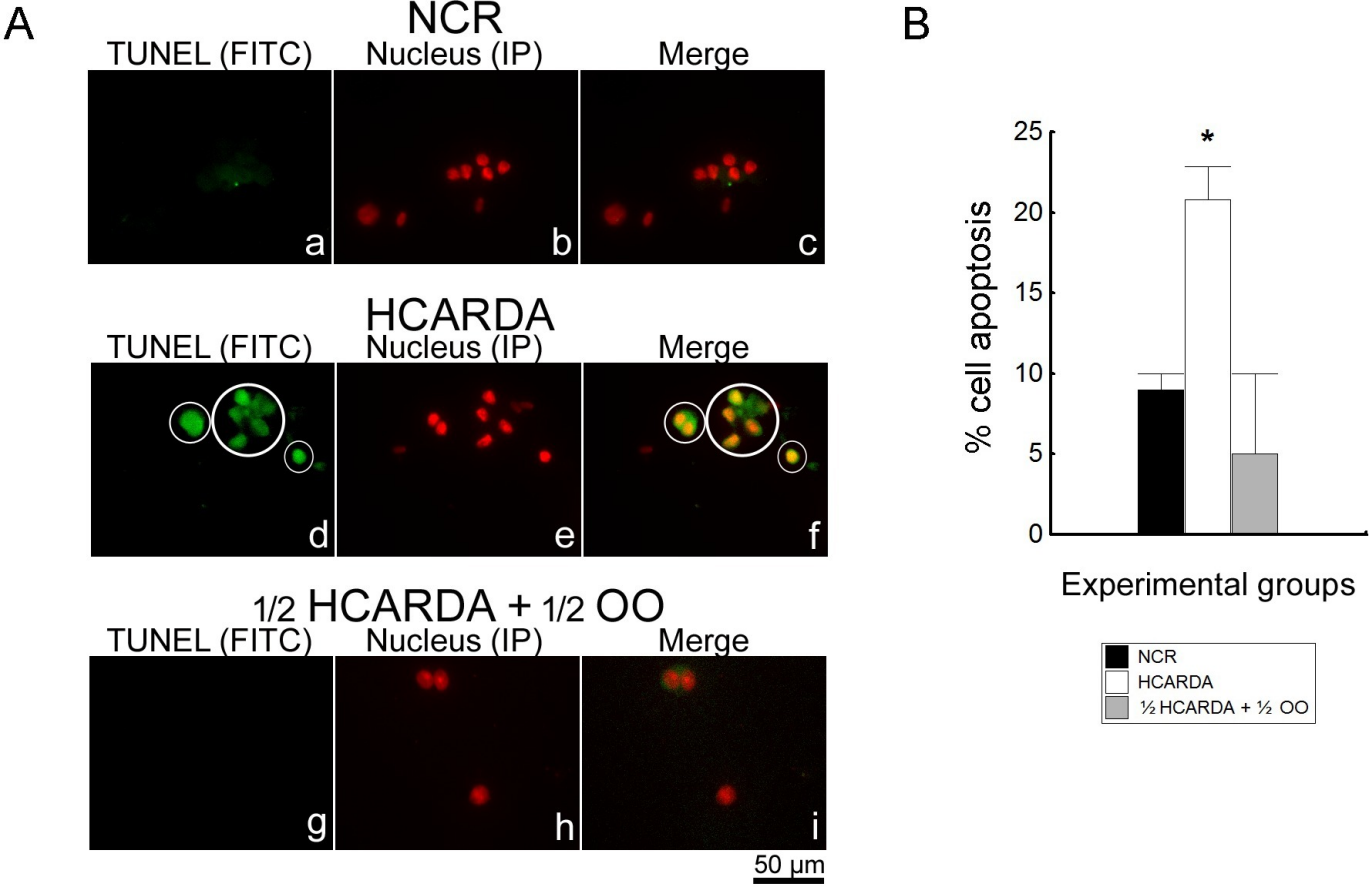
Detection of apoptosis by TUNEL assay in isolated spermatogenic cells. (A) Left column (a, d and g: green signal) corresponds to TUNEL-positive spermatogenic cells, middle column (b, e and h: red signal) to nucleus detection by propidium iodide, and right column (c, f and i) to merge. TUNEL-positive cells are surrounded by a white circle. Magnification: 400X. (B) Mean ± SD of TUNEL-positive cells (%) were plotted for NCR = black bar, HCARDA = white bar, and ½ HCARDA + ½ OO = grey bar. n = 200 cells per experimental condition. Asterisk = *p*< 0.05.

Apoptosis was tested in spermatogenic isolated cells. There was a significant increment in the percentage of TUNEL-positive cells when high-fat diet was implemented (Fig 13, compare HCARDA with NCR). When olive oil was added to the high-fat diet, the percentage of TUNEL-positive cells decreased (Fig 13, ½ HCARDA + ½ OO).

## Discussion

In this paper it was demonstrated that olive oil added to the high-fat diet recovered the normal morphology and count of sperm cells in the semen of hypercholesterolemic rabbits. Previous reports showed that the high-fat diet promotes increased serum cholesterol in rats [25], mice [26] and rabbits [1,2,27]. Hypercholesterolemia is associated with a low number of spermatozoa in semen and abnormal sperm morphology in rabbits [1,20]. It was established that sperm defects are the result of a defective interaction between the manchette-acrosome complex and membrane microdomains [20]. On the other hand, the lower number of sperm can be explained by a decrease in testicular efficiency and an increment in apoptosis of germ cells [20]. Olive oil promoted the normalization of the interaction of the manchette-acrosome complex with lipid microdomains and improved the testicular efficiency with a reduced apoptotic cell count in the testicles.

Hypercholesterolemic animal models were established to study the adverse effect of high cholesterol diets on the structure and function of testes and accessory sexual organs, epididymal maturation of spermatozoa, sperm quality parameters and sperm fertilization capacity [1,28,29]. Our and other authors' studies of the deterioration in semen[2,30,31] that can be improved by adding olive oil to the high-fat diet [6] have shown that cholesterol-enriched diets affect sperm fertilization capacity and embryonic development in rabbits[27].

Cholesterol excess affects several cell types, promoting functional and morphological changes [32]. In spermatozoa, cholesterol accumulates in the acrosome and can interfere with processes such as sperm capacitation and acrosome reaction [6]. In the seminiferous epithelium, the abnormal development of the acrosome associated with changes in the acroplaxome/manchette complex, the presence of cholesterol whorls and several cytoplasmic vacuoles were described [20]. The presence of vacuoles has been previously described in normal rabbits [33], but the type of cell containing these vacuoles was Sertoli cells. Instead, we found lipid droplets and whorls inside the spermatogenic cells. The presence of these structures could explain the alterations in the position of acrosome during the elongation process. With the addition of olive oil to the diet, these alterations were clearly reduced.

In this work we re-examined in more detail the epithelial morphology of rabbit testicles under diets high in fat or protected with olive oil. The altered morphology of spermatozoa in the semen involved an abnormal shape of the head with vesicles and flagella implanted outside the central axis. Sperm head alterations may be attributed to altered function in the testis at different stages during spermiogenesis [20]. In HCARDA, different stages of the seminiferous epithelium could be observed due to the preservation of spermatogenesis. But the last steps of spermiogenesis displayed round nucleus in the apical area of the epithelium and asymmetric sperm heads. This alteration coexisted with a detachment of the seminiferous epithelium. However, when the proportion of fat was reduced (½ HCARDA) the abnormal cells disappeared, but some alterations remained. Spermatogenesis fully recovered only when olive oil was added to the diet.

**Fig 14:**
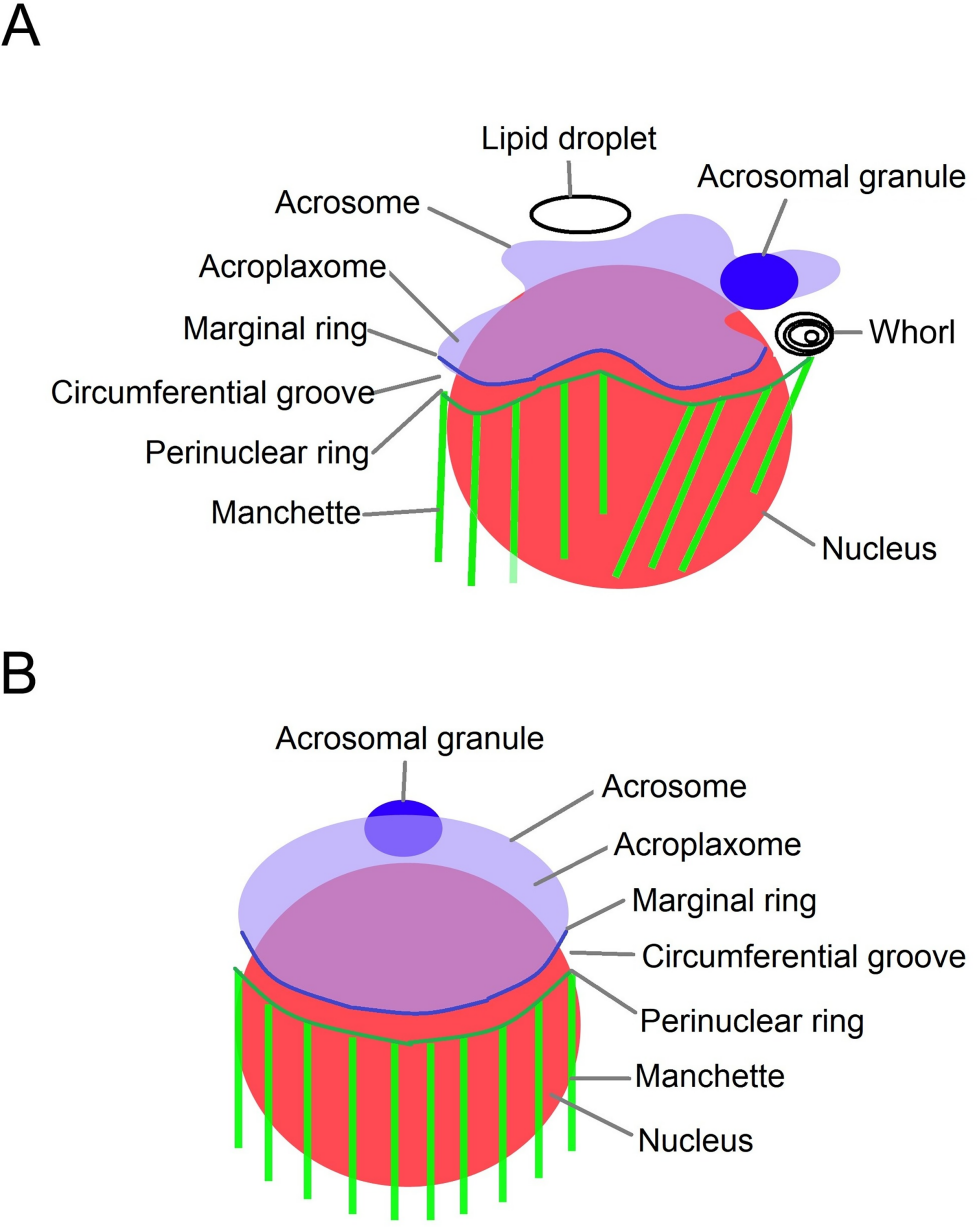
Sperm elongation complex model. The nucleus is represented in red, the acrosome in light blue, the acrosomal granule in blue, the marginal ring with a blue line, the perinuclear ring with a dark green line, the microtubules of manchette with light green lines. The space between acrosome and nucleus corresponds to acroplaxome, and the space between marginal and perinuclear ring to circumferential groove. (A) HCARDA spermatid. The lipid droplet could disturb the acrosomal granule position and modify the elongation axis. The acrosome is lax, the acroplaxome and the marginal ring are disrupted. The whorls in the circumferential groove may affect marginal and perinuclear ring interaction. The manchette seems to pull asymmetrically. (B) ½ HCARDA + ½ OO spermatid. The cytoplasm is free of whorls and lipid droplets. The acrosome granule is positioned equidistantly from both edges of the acrosome. The interaction between marginal and perinuclear rings appears to be recovered. Finally, the acrosome is well positioned on the nucleus and symmetrically pulled by the manchette. This elongation complex is similar to normal.

The asymmetry in the position of the flagella in HCARDA-isolated spermatozoa could be an effect of the asymmetry of the manchette-acrosome complex. The specific fluorescence detection of the main components of the manchette (microtubule hair reaching the marginal zone of the ring, actin filaments that anchor them to the plasma membrane and cholesterol microdomains in the plasma membrane) showed a homogeneous distribution in a large number of cells rather than a location restricted to the manchette zone in HCARDA-spermatids. The acrosome could be observed narrowed to the nucleus and centered to the central axis in spermatids isolated from NCR, but a lax and off-axis acrosome was detected in HCARDA. When dietary fat was reduced, but markedly with the addition of olive oil, these anomalies were reduced. Fig 14 shows a proposed model of the sperm elongation complex in spermatids under high-fat and protected diets.

Analyzing round spermatids in previous stages of spermiogenesis, cytoskeleton structures were observed at acrosomal and manchette zones. The presence of microtubules in the acrosomal zone of NCR and ½ HCARDA + ½ OO (S1 Fig) could demonstrate the existence of an acroframosome-like structure in rabbits, not described previously in mammals [34]. This structure could guide vesicles towards the nucleus during the acrosome morphogenesis [13]. Interested was observing the absence of microtubules at acrosomal zone in HCARDA spermatids (S1 Fig) which could be related with the presence of acrosomal vesicles observed in round spermatids (Fig 9) and sperm cells (Fig 4). Moreover, when we quantified tubulin (S2 Fig), we detected a significant reduction in tubulin expression under high-fat diet. Some authors have demonstrated alterations in sperm heads and nuclear morphogenesis when the tubulin synthesis is blocked [35] which is also observed in HCARDA experimental condition. On the other hand, actin fluorescent intensity was not modified (S1 Fig) demonstrating the conservation of the acroplaxome and related structures in HCARDA.

In accordance with tubulin expression, some authors have described the importance of the microtubules in the transport of residual bodies [36]. There could exist a connection between those phenomena: the residual body relief, the tubulin reduction and the manchette disorders in HCARDA.

Some authors have reported a significant reduction in sperm concentration in different animal models of hypercholesterolemia [28]. The sperm number in semen is related to the performance of the seminiferous epithelium, known as testicular efficiency. In most papers, testicular efficiency has been calculated dividing the number of ejaculated sperm cells by the testicular weight[11,37,38]. However, in our experimental conditions testicular weight did not decrease significantly, which was also reported by other authors (data not shown) [27]. But cholesterol feeding produced a marked decrease in the spermatogenic cell population. In this case, the high-fat diet affected differentiation efficiency (*der* index), demonstrated by a reduction in sperm count, although no significant change in the percentage of spermatids was detected. Moreover, spermatocyte percentage was higher, probably induced by an impediment to the progress of spermatogenesis (similar results have been reported in rats [39,40]. However, differentiation efficiency was restored by adding olive oil to the diet, with an increase in the percentage of spermatids and sperm cells. In this sense, the increment in the percentage of spermatids could be assigned to a reactivation of the flow of the path from spermatogonia to elongated spermatid/sperm. This could be supported by the fact that spermatogonia, spermatocytes and round spermatids percentages were reduced. It could be explained as an improvement in the progress of spermatogenesis induced by the olive oil addition, finally represented in the restoring of the testicular efficiency (*per* and *der* indexes).

Low ejaculated sperm number has been associated with a misbalance between cell survival and apoptosis during spermatogenesis [41]. This misbalance is caused by pathological conditions and environmental factors and can lead to oligospermia [42]. It has been proposed that intracellular lipid increment promotes apoptosis [32]. HCARDA testicles showed lipid increase (lipid droplets and whorls) accompanied by enhanced apoptosis, tested in isolated cells. However, apoptosis was reduced in cells from control and olive oil-protected animals. These overall results, decreased efficiency and increased apoptosis, could explain the reduction in the number of spermatozoa in semen that was restored by the addition of olive oil to the diet.

In conclusion, specific changes in spermatogenesis, that lead to low sperm count and abnormal sperm morphology, were detected in rabbit with high-fat diet induced hypercholesterolemia. Reduced testicular efficiency was associated with decreased progress from germ cells to spermatozoa and increased apoptosis of germ cells. Sperm defects were the result of a defective interaction between the manchette-acrosome complex with membrane microdomains. Interestingly, all these changes, the manchette-acrosome disorders and testicular efficiency decline, were reversed by the diet enriched with olive oil.

## Supporting information

S1 Fig Microtubules and actin filaments arrangement during spermiogenesis under experimental diets with emphasizes at the acrosomal area. (A) Spermatogenic isolated cells were analyzed to test the components of the sperm head cytoskeleton. Microtubules were detected using α tubulin antibody and secondary antibody combined with FITC (tubulin column, a, f and k). Actin filaments were stained with actin antibody conjugated with Cy3 (actin column, b, g and l). Merge of green and red channels (merge column, c, h and m), phase contrast images (DIC column, d, i and n) and toluidine stained cells (stained cells column, e, j and o) were also included. In NCR (first row), microtubules and actin filaments were distributed up (acrosomal zone, az) and down (manchette zone, mz) of the equatorial segment, clearly delimited by perinuclear ring (white arrow in figure e). Acrosomal granule was also detected by DIC (black arrow in figure d). In HCARDA (middle row), fluorescent signals were distributed homogeneously, up and down of nuclear ring. Black arrow mark acrosomal granule position (figure i). In ½ HCARDA + ½ OO (down row), cells recovered the microtubule and actin filaments distribution, over and down the perinuclear ring (white arrow in figures k, m and o). Actin and tubulin signals at the acrosomal zone (az) and manchette zone (mz) were detected. Magnification: 620X.
(TIF)

S2 Fig Tubulin quantification via Western-blot analysis. (A) Tubulin protein detected via Western-blot. Histone H3 was used as a reference protein. Column 1 corresponds to NCR group, column 2 to ½ HCARDA + ½ OO group, and column 3 to HCARDA group. (B) Bars represent mean ± SD of the tubulin protein expression detected via Western-blot. Black bar represents NCR, white bar = HCARDA, and grey bar = ½ HCARDA + ½ OO. n = 3. Asterisks = *p*< 0.05.
(TIF)
