# Supplementary figures and images for: Manchette-acrosome disorders and testicular efficiency decline observed in hypercholesterolemic rabbits are recovered with olive oil enriched diet
Source: PLoS One. 2018 Aug 23;13(8):e0202748. doi: 10.1371/journal.pone.0202748 (PMC6107225; doi:10.1371/journal.pone.0202748)

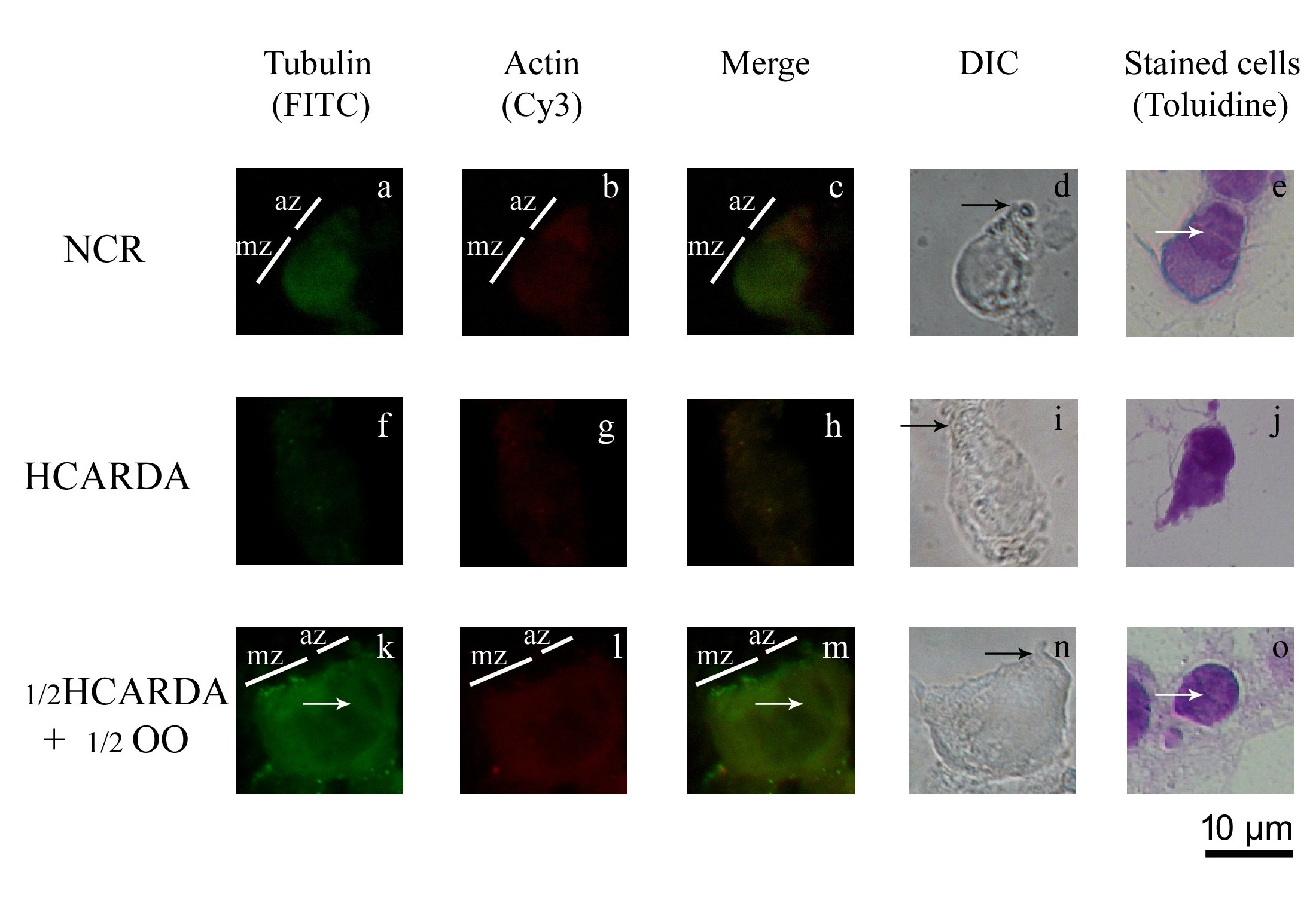

Supplement: S1 Fig — (A) Spermatogenic isolated cells were analyzed to test the components of the sperm head cytoskeleton. Microtubules were detected using α tubulin antibody and secondary antibody combined with FITC (tubulin column, a, f and k). Actin filaments were stained with actin antibody conjugated with Cy3 (actin column, b, g and l). Merge of green and red channels (merge column, c, h and m), phase contrast images (DIC column, d, i and n) and toluidine stained cells (stained cells column, e, j and o) were also included. In NCR (first row), microtubules and actin filaments were distributed up (acrosomal zone, az) and down (manchette zone, mz) of the equatorial segment, clearly delimited by perinuclear ring (white arrow in figure e). Acrosomal granule was also detected by DIC (black arrow in figure d). In HCARDA (middle row), fluorescent signals were distributed homogeneously, up and down of nuclear ring. Black arrow mark acrosomal granule position (figure i). In ½ HCARDA + ½ OO (down row), cells recovered the microtubule and actin filaments distribution, over and down the perinuclear ring (white arrow in figures k, m and o). Actin and tubulin signals at the acrosomal zone (az) and manchette zone (mz) were detected. Magnification: 620X. (TIF) [file pone.0202748.s001.tif]

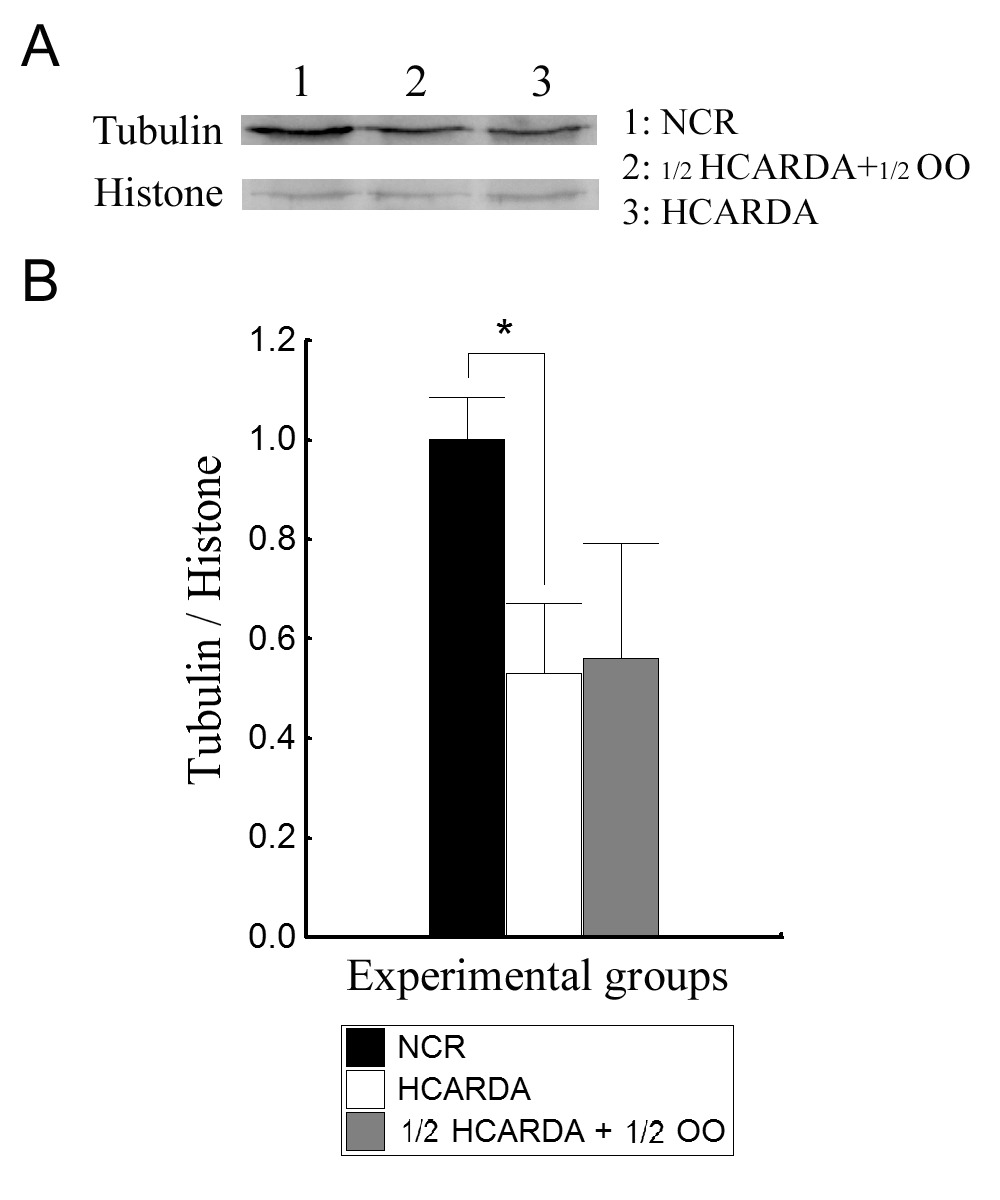

Supplement: S2 Fig — (A) Tubulin protein detected via Western-blot. Histone H3 was used as a reference protein. Column 1 corresponds to NCR group, column 2 to ½ HCARDA + ½ OO group, and column 3 to HCARDA group. (B) Bars represent mean ± SD of the tubulin protein expression detected via Western-blot. Black bar represents NCR, white bar = HCARDA, and grey bar = ½ HCARDA + ½ OO. n = 3. Asterisks = p< 0.05. (TIF) [file pone.0202748.s002.tif]
